# Successful osimertinib rechallenge following drug-induced pneumonitis after previous anti-PDL1 exposure

**DOI:** 10.3332/ecancer.2019.970

**Published:** 2019-10-21

**Authors:** Guilherme Harada, Fernando Costa Santini, Felipe Sales Nogueira Amorim Canedo, Leandro Jonata de Carvalho Oliveira, Henrique Bortot Zuppani, Gilberto De Castro

**Affiliations:** 1 Hospital Sírio Libanês, São Paulo 01525-001, Brazil; 2 Instituto do Câncer do Estado de São Paulo, São Paulo 01525-001, Brazil

**Keywords:** lung cancer, osimertinib, immunotherapy, pneumonitis, rechallenge, brain metastases

## Abstract

Osimertinib is a first-line treatment option for patients with metastatic non-small cell lung cancer (NSCLC) harbouring EGFR mutations. Pneumonitis is a severe adverse event (AE) related to osimertinib treatment which appears to be more frequent when associated with concurrent or previous anti-PD(L)1 exposure. Data regarding the efficacy and safety of osimertinib rechallenge, especially in the setting of central nervous system (CNS) metastases, are scarce. We herein describe a case of a 53-year-old patient with metastatic EGFR-mutated NSCLC, who developed pneumonitis after osimertinib treatment and was successfully rechallenged with 40 mg daily osimertinib, with CNS response. This dose reduction strategy may be an option for selected patients with brain metastases after tyrosine kinase inhibitors-induced AEs.

## Introduction

Brain metastases (BMs) occur initially in 20% of patients with non-small cell lung cancer (NSCLC) and 30%–50% of patients will be diagnosed with BMs during the course of their disease [1, 2]. In patients with EGFR-mutated tumours, the incidence of BMs at the time of diagnosis is higher than in unselected patients (25%), suggesting that EGFR mutations might be associated with a metastatic tropism to the brain [3].

Among patients with NSCLC with EGFR mutations, tyrosine kinase inhibitors (TKIs) seem effective in controlling the intracranial disease. Preclinical and clinical evidence indicate that EGFR-TKIs of the third generation are more effective in treating BMs than their first- and second-generation counterparts, showing a promising blood–brain barrier (BBB) penetration and the potential to overcome EGFR-TKI resistance [4].

Lung abnormalities, from transient asymptomatic pulmonary opacities to full-blown interstitial lung disease, have been described as adverse events (AEs) associated with osimertinib, a third-generation EGFR-TKI. Severe AEs have been reported when EGFR-TKIs (especially osimertinib) follow immunotherapy [5]. Potential interactions of these therapies can cause unpredicted overlapping AEs.

We hereby describe a case of successful half-dose osimertinib rechallenge with central nervous system (CNS) response in a patient with *EGFR*-mutated NSCLC, previously treated with an anti-PD-L1 antibody, which developed severe pneumonitis after initial osimertinib exposure.

## Case report

**Figure 1.**
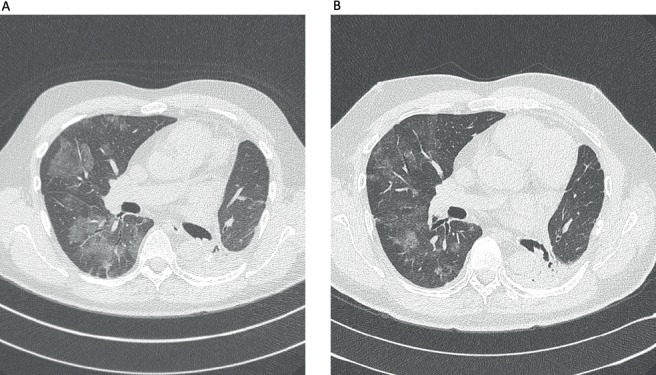
Chest CT findings before and after corticotherapy. (A): Patchy ground-glass opacity, mainly in the right lung. (B): Significant improvement after 3 weeks with prednisone.

**Figure 2.**
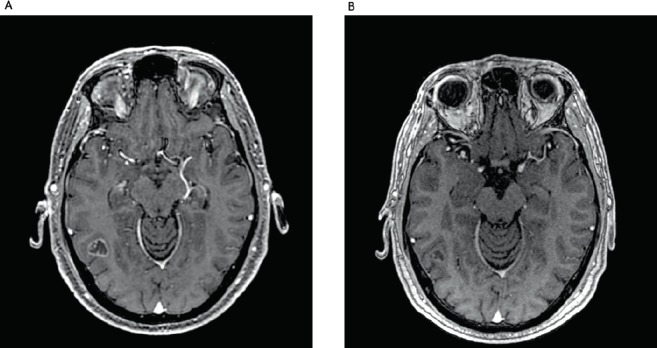
(A): Brain MRI demonstrating right occipitotemporal lesion measuring 1.8 cm × 1.4 cm. (B): Two months after rechallenge of osimertinib and reduction of the lesion, measuring 0.8 cm × 0.5 cm.

Here, we report a 53-year-old male, never-smoked patient, who had undergone left lower lobectomy and mediastinal sampling for lung adenocarcinoma, pathologic stage IIIC (T3N3M0), harbouring an *EGFR* exon 19 deletion (p.Leu747_Ala750delinsPro; NM_00528), detected by pyrosequencing (PyroMark Q24, Qiagen, Tokyo, Japan). Post-surgical 18-fluorodeoxyglucose positron emission tomography/computerized tomography revealed positive upper and lower contralateral (right) paratracheal nodes. Definitive concurrent chemoradiation (54 Gy delivered concurrently with cisplatin 50 mg/m^2^ on days 1, 8, 29 and 36, plus etoposide 50 mg/m^2^ daily on days 1–5 and 29–33) was administered up to February 2018, followed by durvalumab 10 mg/kg, starting in April 2018. After eleven 14-day cycles, he complained of a new-onset headache. Brain MRI revealed widespread supra and infratentorial brain parenchymal metastasis, and he underwent surgical resection of two bilateral frontal metastases (left 4.1 cm and right 2.8 cm), which confirmed *EGFR*-mutant lung adenocarcinoma, harbouring the same *EGFR* exon 19 deletion (p.Leu747_Ala750delinsPro; NM_00528). Twenty-two days after durvalumab interruption, osimertinib 80 mg once daily was initiated. On the 53rd day of osimertinib treatment, the patient was admitted due to intense dyspnoea on exertion and cough. Chest CT scan revealed patchy ground-glass opacities (Figure 1). As interstitial lung disease induced by osimertinib was considered, EGFR-TKI was suspended and prednisone 1 mg/kg/day was started, along with piperacillin-tazobactam. Three weeks later, a new CT scan showed significant improvement and all symptoms subsided. Patient was rechallenged with osimertinib 40 mg, 42 days after its suspension, and prednisone was rapidly tapered down. As of today, 2 months after this rechallenge, new brain MRI showed tumour response, including shrinkage in the right occipitotemporal lesion (0.8 cm × 0.5 cm; previously 1.8 cm × 1.4 cm) (Figure 2) and the left cerebellar lesion (0.5 cm; previously 1.1 cm × 0.8 cm). No signs of pneumonitis recurrence were noticed.

## Discussion

Osimertinib is a treatment option in the first-line setting for patients with metastatic *EGFR*-mutated NSCLC [6]. It achieves greater intracranial concentrations than first- and second-generation EGFR-TKIs [7] and exhibited a longer progression-free survival (PFS) (15.2 months versus 9.6 months; hazard ratio 0.47, 95% confidence interval (CI) 0.30–0.74) in a subset analysis of the FLAURA trial, which included 116 treatment-naïve patients with *EGFR-*mutated advanced NSCLC and CNS metastases [8]. In the FLAURA trial, osimertinib showed a lower rate of CNS progression (6% versus 15%) and an increased intracranial response rate (91% versus 68%) than first-line treatment with gefitinib or erlotinib [6].

In order to optimise the CNS control in *EGFR*-mutated patients, the combination of targeted therapies and radiotherapy has been studied. Radiotherapy is known to disrupt BBB and potentially increases the effect of EGFR-TKIs in CNS, even if such inhibitors already penetrate the BBB [2]. A retrospective multi-institutional analysis suggested that stereotactic radiosurgery (SRS) followed by first-generation EGFR-TKI resulted in longer overall survival (OS) when compared with EGFR-TKI followed by SRS or whole-brain radiotherapy at progression [9]. Regarding the association of osimertinib and radiotherapy, Xie *et al* did not demonstrate superiority in time to treatment failure, PFS and OS of association of radiotherapy and osimertinib compared to osimertinib alone in a retrospective analysis [10]. Osimertinib has not been evaluated with SRS in prospective trials yet, and additional studies are needed to address these questions. A clinical trial is open to evaluate osimertinib with or without SRS for *EGFR*-mutated NSCLC with BM (NCT03497767).

The increase of osimertinib toxicity in patients who used anti-PD(L)1 was recently described, and interstitial lung disease is one of the main AEs [5]. The concomitant or sequential use of osimertinib and durvalumab was also associated with a high risk of pneumonitis [11, 12], confirming that this association is related to a higher risk of immunotoxicity. It is important to highlight that this overlapping toxicity seems to be osimertinib-related, rather than class-specific, since the association of other EGFR-TKIs and anti-PD(L)1 did not present the same toxicity rates [13].

Osimertinib rechallenge is an important point to be considered. A successful full-dose osimertinib rechallenge has been reported in a patient with BMs following salvage cytotoxic chemotherapy, showing potential intracranial therapeutic effect with re-exposure to the drug [14]. Metro *et al* described a case of an *EGFR* T790M-positive lung cancer patient, who was pretreated with the sequence erlotinib–osimertinib and experienced a dramatic response to osimertinib rechallenge after intervening chemotherapy [15]. In a retrospective analysis, 17 patients were rechallenged with osimertinib after acquiring resistance. The objective response was 33% and disease control rates were 73%. The median PFS was 4.1 months (95% CI: 1.9–6.7). The toxicity was low, being that most patients had grade 2 adverse events (AEs) or lower, without interruption of treatment due to AEs [16].

Previous experiences indicate that re-administration of 40 mg osimertinib may be a safe and effective strategy in patients who developed osimertinib-induced pneumonitis with standard-dose use [17]. Clinicians must be aware of osimertinib potential toxicities and of viable strategies to manage them so as to guarantee maximum benefit to metastatic *EGFR*-mutated NSCLC patients. The present case indicates that it may be feasible to treat patients with BMs with osimertinib 40 mg daily if dose reduction is needed after a severe drug-related AE.

## Conclusion

To the authors’ knowledge, this is the first report of activity with CNS response and safety after rechallenge with osimertinib 40 mg. The safest time interval between interrupting immunotherapy and starting osimertinib is still an unanswered question. Once patients with BMs have the urgency to start a CNS active therapy and considering the efficacy of osimertinib in *EGFR*-mutant NSCLC, osimertinib rechallenge can be considered in selected cases.

## Conflicts of interest

Dr de Castro Junior reports personal fees and other from AstraZeneca, personal fees and other from Roche, personal fees and other from Boehringer-Ingelheim, outside the submitted work.

The other authors declare that they have no conflicts of interest.

## Funding statement

No funding was received.
